# Comparison of efficacy and safety of laparoscopic excision and open operation in children with choledochal cysts: A systematic review and update meta-analysis

**DOI:** 10.1371/journal.pone.0239857

**Published:** 2020-09-28

**Authors:** Rui Sun, Na Zhao, Ke Zhao, Zhe Su, Yifan Zhang, Mei Diao, Long Li

**Affiliations:** 1 Department of Pediatric Surgery, Capital Institute of Pediatrics, Beijing, China; 2 Chinese Academy of Medical Sciences and Peking Union Medical College, Beijing, China; 3 Department of Radiology, Fuwai Hospital, State Key Laboratory of Cardiovascular Disease, National Center for Cardiovascular Diseases, Beijing, China; 4 Department of Ophthalmology, Ningbo Hangzhou Bay Hospital, Ningbo, China; Zagazig University, EGYPT

## Abstract

**Objective:**

The outcomes of children with Choledochal cyst who undergo laparoscopic cyst excision and Roux-en-Y hepaticojejunostomy versus open cyst excision and Roux-en-Y hepaticojejunostomy have not been adequately compared. We conducted a systematic review and meta-analysis to gain further insight into the efficacy and safety of laparoscopic excision in children with choledochal cysts.

**Methods:**

A systematic search of PubMed, Embase, Cochrane Central Register, and ClinicalTrials.gov databases from January 1973 to January 31, 2020 was performed utilizing the PRISMA guidelines. Short-term, long-term and total postoperative complications were the primary endpoint measurements, whereas intraoperative outcomes and other postoperative outcomes were the secondary endpoints.

**Results:**

The final analysis included 14 retrospective cohorts comprising 1767 patients. There were no significant differences in the patients’ short-term postoperative complications (RR = -1.08; 95% CI = -1.72 to -0.67) between the 2 approaches. However, improvements in long-term (RR = 0.09; 95% CI = 0.01 to 0.18) and total postoperative complications (RR = -0.29; 95% CI = -0.40 to -0.21), estimated intraoperative blood loss and transfusion, time of initial feeding, and length of hospital stay were observed in patients who underwent laparoscopic excision when compared to those who underwent open surgery.

**Conclusions:**

Laparoscopic cyst excision and Roux-en-Y hepaticojejunostomy provides similar or even improved intraoperative, postoperative outcomes when compared to open excision for children with Choledochal cyst.

## Introduction

Choledochal cyst (CDC) is a rare congenital malformation of the biliary system. It was initially described by Vater in 1723 and classified by Todani et al [1] in 1977. The incidence is around 1:15,000 live births [2, 3]. CDC is more common in East Asian nations [4] and affects girls more than boys [5]. Although CDC can be diagnosed at all stages, it is primarily seen in children. Generally, CDC requires surgical intervention in order to avoid complications such as cholangitis, perforation, liver failure and even malignancy. Currently, complete cyst excision with cholecystectomy followed by biliary reconstruction using a Roux-en-Y hepaticojejunostomy is the standard treatment of choice [6]. This surgery is a complex procedure in biliary tract surgery; therefore, it is used to perform using an open operation. However, open excision for children with choledochal cysts requires a generous incision of the abdominal wall for hepatojejunostomy.

The first successful laparoscopic choledochal cyst excision and hepaticojejunostomy was performed on a 6-year-old girl in 1995 by Farello [7]. Since then, laparoscopic excision has been increasingly adopted as a viable surgical treatment option of CDC. This technique includes many advantages, including minimal scar, and a clear and magnified view, which can significantly facilitate the accuracy of dissection and anastomosis. Comparing with the conventional open surgery for children with CDC, the advantages of laparoscopic excision are already well documented [8], including less surgical trauma, less bleeding, and smaller scars. With the rapid development of laparoscopic techniques in recent years, laparoscopy excision in children with CDC have evolved at an unprecedented pace. Nonetheless, the laparoscopic choledochal cyst surgery in children is a procedure with more technical challenge and complexity [9]. Two published in 2014 [10, 11] had confirmed an improvement in some perioperative outcomes in children with CDCs through laparoscopic excision; however, they failed to reveal the rate of postoperative complications between 2 approaches since insufficient evidence. Therefore, the safety and efficacy of laparoscopic procedure remain controversial. Moreover, there were many relevant studies published since 2014, which were not included in previous studies. Considering that postoperative complications may seriously affect the growth and development of children; thus, we performed an update systematic review and meta-analysis focusing with the primary results being short-term, long-term, and total postoperative complications, and secondary results being perioperative outcomes comparing those who received laparoscopic excision with open excision for children with CDCs.

## Materials and methods

This study was performed in accordance with the recommendations of the Preferred Reporting Items for Systematic Reviews and Meta-analyses (PRISMA) reporting guideline [12]. We have registered our study on the PROSPERO, of which ID is CRD42019137474 (S1 File).

### Data sources and searches

We systematically searched databases, including PubMed, Embase, the Web of Science, Cochrane Library and ClinicalTrials.gov for studies published between 1973 to February, 2020. The search Medical Subject Heading (MeSH) terms were “choledochal cyst” AND “laparoscopic”, as well as all associated entry words retrieved using the MeSH index (details of our search strategy are included in S2 File). The language was restricted to English only. We also reviewed the introduction and discussion sections of retrieved trials, relevant review articles, and published meta-analysis to identify additional trials. Two of us (RS and NZ) independently conducted the literature search, screening of abstracts, and selection of included trials.

### Inclusion criteria

The studies that published up to and included between January 1973 and February 2020 were considered eligible if they met the following inclusion criteria: (1) population: children younger than 18 years with choledochal cysts; (2) intervention: laparoscopic cyst excision and Roux-en-Y hepaticojejunostomy (LA); (3) comparison: open cyst excision and Roux-en-Y hepaticojejunostomy (OP); (4) outcomes: study reported on at least one of the outcome measures mentioned below: operative time, estimated intraoperative blood loss (EIBL), intraoperative blood transfusion, initial feeding, length of hospital stay (LOS), postoperative morbidity and mortality.

### Exclusion criteria

The exclusion criteria are the following: (1) review articles; (2) meeting abstracts; (3) studies that only include 1 surgical technique; (4) studies with no comparative data; (5) full text not in English or insufficient information available in English abstract; (6) not the relevant studies; (7) the population is adult; (8) if papers had overlapping data, those describing the smaller-scale studies were excluded.

### Quality assessment

We adopted the Newcastle–Ottawa Scale (NOS) [13], which is designed specifically for observational investigations, to assess the quality of the selected studies. The NOS focuses on 3 separate sections of a case-control or cohort study, with the number of stars representing the assessment score. The maximum achievable score under the NOS is 9 stars, including 4 for the selection process, 2 for comparability, and 3 for exposure and outcome. A score of ≥6 stars is considered indicative of high quality. Two investigators independently assessed the selected studies.

### Data extraction

Two investigators independently extracted the following information: first author, year of publication, study type, mean age, number of population, and main outcomes, including operative time, EBIL, intraoperative blood transfusion, initial feeding, LOS, postoperative complications. The evaluators resolved any disputes via consensus during the screening processes.

### Statistical analysis

Statistical analyses were conducted using Review Manager 5.3 (Cochrane Collaboration). The relative risk (RR) and mean difference (MD) with the 95% confidence interval (CI) were used as the measures of dichotomous and continuous variables, respectively. Some studies only reported outcomes of medians with ranges and mid-quartile with ranges; therefore, according to methods introduced by Wan et al. [14] and Luo et al. [15], medians with ranges and mid-quartile with ranges were converted into means with standard deviations. Heterogeneity was considered not statistically significant when the Cochrane Q test P value was >0.1 or the value of Q was < 50%. A transformation of Q test, the I^2^ statistic (I^2^ = 100% × (Q − df)/Q), was used to assess the consistency of the effect sizes. Therefore, a study with an I^2^ less than 50% was considered as low heterogeneity, and greater than 50% as high heterogeneity. The fixed effect model was used to combine the data in case of the absence of heterogeneity between studies, and the random-effect model was used when heterogeneity was present. To assess the effects of any single study, sensitivity analysis was conducted. The Grading of Recommendations Assessment, Development, and Evaluation (GRADE) approach is used to evaluate the quality of the evidence. The evidence was categorized as high, moderate, low or very low quality [16]. The criteria for the evaluation of the evidence included the assessment of the risk of bias as determined by the GRADEpro (https://gdt.gradepro.org), which is an online and free APP. Publication bias was assessed using the asymmetry of the Funnel. P values less than 0.05 indicated statistical significance.

## Results

### Search results and characteristics of included studies

**Fig 1:**
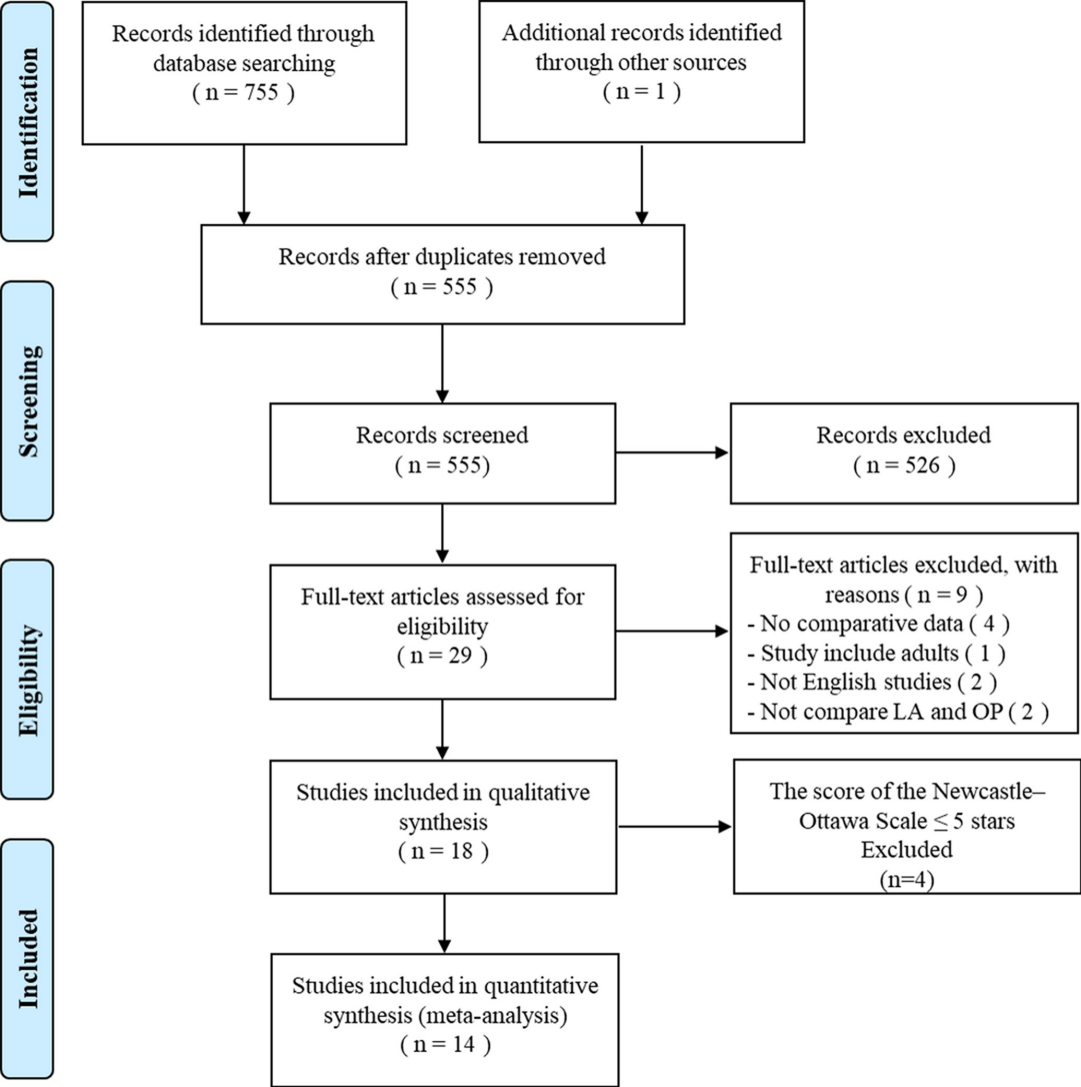
Flowchart showing the protocol of the meta-analysis.

A total of 756 studies were found in the primary literature search. After excluding duplicate studies and carefully reviewing the title, abstract and full text, there were 18 studies that compared LA and OP for children with children. Of the above 18 studies, 4 studies were further excluded due to the results of NOS, with score being ≤ 5 stars, which were considered as indicative of low quality. Finally, in total, our analysis included 14 [14–27] retrospective cohort studies comparing LA and OP in the children with CDCs, with 1767 patients (853 in the LA group, 914 in the OP group) enrolled. A flowchart of our analysis protocol has shown in Fig 1. The characteristics and the quality of these 14 studies are listed in Table 1. The extracted results of each enrolled studies were showed in the Table 2. The means of the patients’ ages in each study ranged from 7 days to 18 years with a majority in both the LA and OP cohorts being of comparable age, only patients of both groups in YU et al.’s study [17] (5.6 ± 3.3 versus 5.9 ± 3.5 years) were older than those in other studies. Table 3 showed the summary of the findings of the GRADE approach.

**Table 1:** The characteristics and qualities of 14 studies in the meta-analysis.

Author, year	Study type	Number of patient LA OP		Sex(M/F) LA OP		Age (day, month, year) LA OP		Follow-up(months) LA OP		NOS
She [30], 2009	Retrospective	10	65	NM	NM	45m (0-16y)	45m (0-16y)	62		7
Diao [28], 2011	Retrospective	218	200	56/162	51/149	4.16m (7d-18y)	4.59y (13d-17y)	38	146	8
Liem [18], 2011	Retrospective	309	307	NM	NM	48.7 ± 2.3m	63.5 ± 2.9m	NM	NM	7
Huang [19], 2011	Retrospective	39	38	9/30	8/30	5y (3m-13y)	4y (2m-15y)	36		8
Cherqaoui [20], 2012	Retrospective	10	9	1/8	3/7	53.7m	62.5m	8.11	67.8	7
Ng [29], 2014	Retrospective	13	22	8/5	3/19	36.5 m	36.5m	35	41	8
Tang [21], 2015	Retrospective	7	5	NM	NM	3.83 ±3.04m	3.83 ± 3.04m	6–12		6
Dalton [22], 2016	Retrospective	11	7	1/10	0/7	3.4 ± 4.1y	6.0 ± 5.8y	37.2		6
Matsumoto [23], 2016	Retrospective	6	7	2/4	2/5	152 (20–268)d	34 (8–550)d	33	146	8
YU [17], 2016	Retrospective	70	86	39/31	42/44	5.6 ± 3.3y	5.9 ± 3.5y	NM	NM	6
Miyano [24], 2017	Retrospective	27	31	4/23	6/25	38.5 (2–123)m	42.2 (1–190)m	4.8	9.9	6
Song [25], 2017	Retrospective	102	104	24/78	30/74	33.5 ± 28.3m	42.4 ± 34.2m	54		8
Urushihara [26], 2018	Retrospective	10	11	4/6	2/9	117(20–268)d	39(8–270)d	37.2	175.2	7
Ruy [27], 2019	Retrospective	22	21	3/19	4/17	14 (7–22)d	13 (9.5–21)d	NM	NM	6

LA, laparoscopic cyst excision and Roux-en-Y hepaticojejunostomy; OP, open cyst excision and Roux-en-Y hepaticojejunostomy; M, male; F, female; NM, not mention, d, day; m, month; y, year.

**Table 2:** Extracted outcomes of the enrolled studies for LA and OP in this meta-analysis.

Study	Samples	Operative time (minutes)	EIBL (ml)	Intraoperative	Initial feeding (Days)	LOS (Days)	Short-term PC	Long-term PC	Total PC
		Mean ± SD	Mean ± SD	blood transfusion	Mean ± SD	Mean ± SD			
She [30], 2009	LA:10	Not mention	Not mention	Not mention	Not mention	Not mention	1	2	3
	OP:65						3	10	13
Diao [28], 2011	LA:218	Not mention	9.08 ± 6.13	0	2.86 ± 1.23	7.41 ± 2.39	Not mention	Not mention	6
	OP:200		35.33 ± 33.29	16	3.78 ± 1.52	9.94 ± 3.47			82
Liem [18], 2011	LA:309	182.7 ± 22.13	Not mention	10	2.5 ± 0.1	7.0 ± 0.2	12	Not mention	12
	OP:307	156.9 ± 8.25		34	3.7 ± 0.1	9.1 ± 0.2	17		17
Huang [19], 2011	LA:39	241 ± 52	14 ± 11.8	0	3.5 ± 0.7	5.5 ± 0.9	6	2	8
	OP:38	190 ± 31	72 ± 110	3	4.9 ± 0.9	7.0 ± 1.4	4	2	6
Cherqaoui [20], 2012	LA:10	288.56 ± 88.68	Not mention	Not mention	3.33 ± 1.67	12.67 ± 9.04	1	Not mention	1
	OP:9	206 ± 40.41			2.5 ± 0.65	7.9 ± 0.65	3		1
Ng [29], 2014	LA:13	Not mention	Not mention	Not mention	3.25 ± 0.3	10.25 ± 6.28	1	0	1
	OP:22				3.25 ± 0.79	5.5 ± 0.52	0	7	7
Tang [21], 2015	LA:7	327.14 ±70.17	Not mention	Not mention	Not mention	14.43 ± 4.65	Not mention	Not mention	0
	OP:5	276 ± 71.62				13.6 ± 2.19			0
Dalton [22], 2016	LA:11	330 ± 42	10 ± 5.7	Not mention	3.5 ± 1.3	5.5 ± 2.2	Not mention	Not mention	1
	OP:7	348 ± 132	121 ± 299		4 ± 1.2	6.9 ± 1.9			2
Matsumoto [23], 2016	LA:6	400 ± 78.03	4.5 ± 2.34	Not mention	3.25 ± 0.39	11.25 ± 1.95	0	0	0
	OP:7	297.75 ± 38.48	34.25 ± 23.09		6.5 ± 2.2	18.5 ± 4.4	0	2	2
YU [17], 2016	LA:70	Not mention	234 ± 45	Not mention	Not mention	Not mention	Not mention	Not mention	5
	OP:86		456 ± 63						16
Miyano [24], 2017	LA:27	413 ± 90.15	Not mention	Not mention	Not mention	Not mention	3	2	5
	OP:31	344.25 ± 45.04					1	3	4
Song [25], 2017	LA:102	225.4 ± 51.0	12.9 ± 22.9	1	3.3 ± 0.9	7.5 ± 2.7	8	2	10
	OP:104	170.3 ± 35.4	32.4 ± 52.7	7	4.1 ± 0.9	9.6 ± 5.5	8	12	20
Urushihara [26], 2018	LA:10	360 ± 93.75	10 ± 18.1	Not mention	3 ± 1.29	10.5 ± 7.76	1	0	1
	OP:11	310 ± 58.37	30 ± 21.03		6 ± 2.2	18 ± 5.65	0	3	3
Ruy [27], 2019	LA:22	235.0 ± 47.2	Not mention	0	4.4 ± 2.1	10.8 ± 5.0	Not mention	Not mention	0
	OP:21	208.3 ± 71.0		2	4.2 ± 1.2	9.9 ± 5.9			5

LA, laparoscopic cyst excision and Roux-en-Y hepaticojejunostomy; OP, open cyst excision and Roux-en-Y hepaticojejunostomy; EIBL, estimated intraoperative blood loss

LOS, length of hospital stay; PC, postoperative complication

**Table 3:** Summary of findings according to GRADE.

Outcome	NO. of studies	Study design	Certainty assessment					Certainty	Importance
			Risk of bias	Inconsistency	Indirectness	Imprecision	Other considerations		
Operative time	10	Observational study	Not serious	Not serious	Not serious	Not serious	Publication bias strongly suspected	⨁⨁⨁◯	Important
							Very strong association	MODERATE	
Estimated intraoperative blood loss	7	Observational study	Serious	Not serious	Not serious	Not serious	Publication bias strongly suspected	⨁⨁◯◯	Important
							Very strong association	LOW	
Intraoperative blood transfusion	5	Observational study	Not serious	Not serious	Not serious	Not serious	Strong association	⨁⨁⨁◯	Important
								MODERATE	
Initial feeding	10	Observational study	Not serious	Not serious	Not serious	Not serious	Publication bias strongly suspected	⨁◯◯◯	Important
								VERY LOW	
Length of hospital stay	11	Observational study	Not serious	Not serious	Not serious	Not serious	Publication bias strongly suspected	⨁⨁◯◯	Important
							Strong association	LOW	
Short-term postoperative complications	9	Observational study	Not serious	Not serious	Not serious	Not serious	Strong association	⨁⨁⨁◯	Critical
								MODERATE	
Long-term postoperative complications	7	Observational study	Not serious	Not serious	Not serious	Not serious	Strong association	⨁⨁⨁◯	Critical
								MODERATE	
Total postoperative complications	14	Observational study	Not serious	Not serious	Not serious	Not serious	Strong association	⨁⨁⨁◯	Critical
								MODERATE	

### Operative time

**Fig 2:**
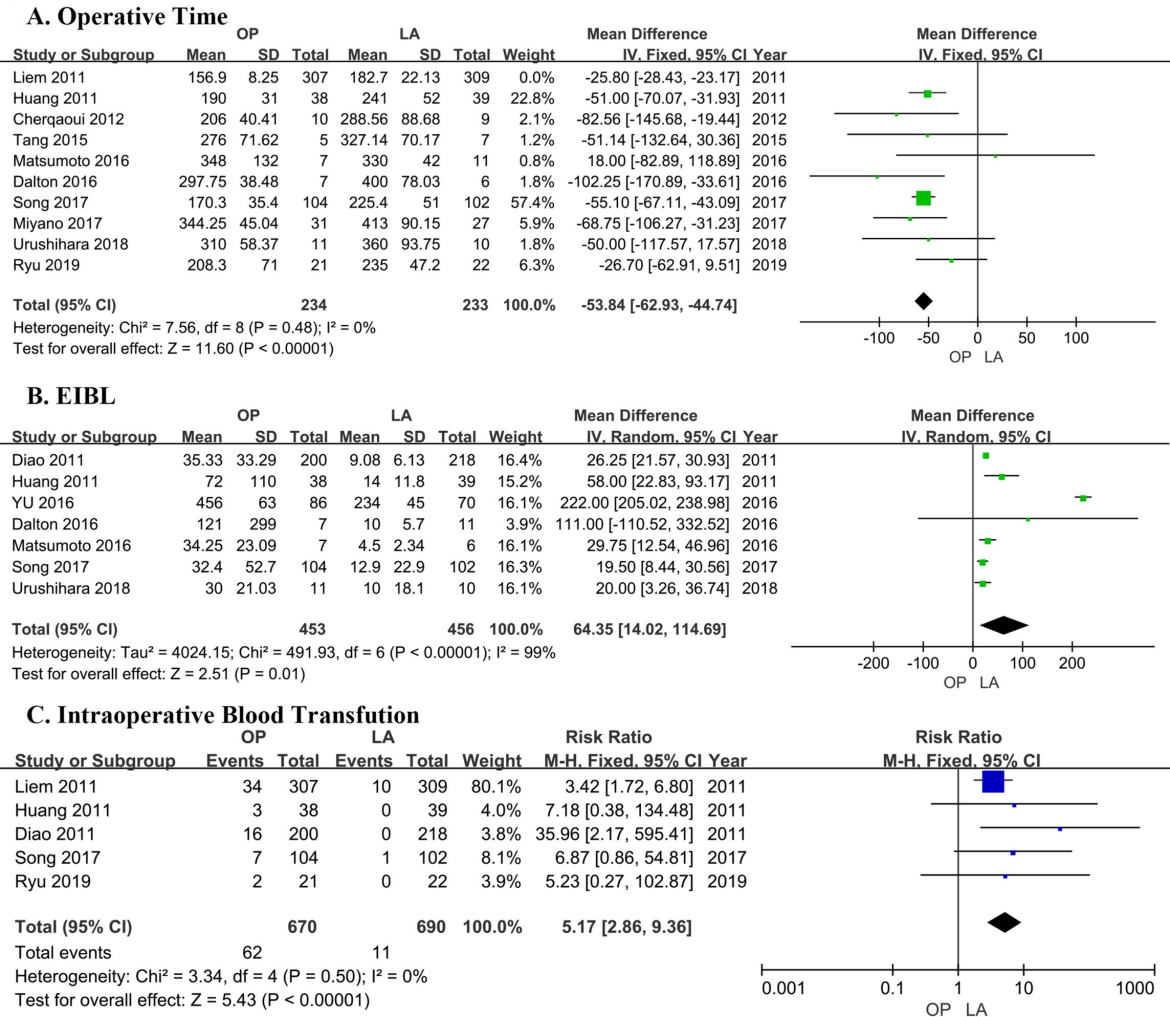
Comparison of the intraoperative outcomes of patients who underwent laparoscopic cyst excision and Roux-en-Y hepaticojejunostomy (LA) and open cyst excision and Roux-en-Y hepaticojejunostomy (OP). (A) Operative time. (B) Estimated intraoperative blood loss (EIBL). (C) Intraoperative blood transfusion.

Ten trials [18–27] contributed data, including a total of 1083 patients (542 in LA, 541 in OP). All studies showed the duration of operation was longer in the laparoscopic group than in the open group. The pooled estimates of those studies showed that the operative time was longer in the LA group. Pooled mean difference (MD = -53.84 minutes; 95% CI = -62.93 to -44.74 minutes; P<0.00001) indicated that the difference is statistically significant. The analysis found statistically significant heterogeneity (P<0.00001), which was high (I^2^ = 78%), then a random-effect model was adopted (Fig 2A). After excluding the study of Liem et al’s [18], the heterogeneity was resolved (P = 0.48, I^2^ = 0%) (S1 Fig) and the mean difference was also changed (MD = -48.13 minutes; 95% CI = -65.37 to -30.88 minutes; P<0.00001).

### EIBL

Seven studies [17, 19, 22, 23, 25, 26, 28] compared the intraoperative bleeding, including a total of 909 patients (456 in LA, 453 in OP). The intraoperative bleeding in the LA group was less than that in the OP group. Pooled mean difference (MD = 64.35ml; 95% CI = 14.02 to 114.69 ml; P = 0.01) indicated that the difference is statistically significant (Fig 2B). There is a significant heterogeneity present in the trials (P<0.00001, I^2^ = 99%), a random effect model was considered. A sensitivity analysis was conducted by excluding YU et al [17], the heterogeneity was solved (P = 0.33, I^2^ = 13%) and the mean difference became 25.44 ml, 95% CI from 20.01 to 30.86 ml (S2 Fig).

### Intraoperative blood transfusion

Five studies [18, 19, 25, 27, 28] compared the intraoperative blood transfusion, including a total of 1360 patients (690 in LA, 670 in OP, Table 2). The pooled results showed a higher rate of intraoperative blood transfusion in the OP group. Pooled RR (RR = -0.19; 95% CI = -0.35 to -0.11; P< 0.00001) showed statistical difference of intraoperative blood transfusion between the 2 groups. Heterogeneity was not significant (I^2^ = 0%) (Fig 2C).

### Initial feeding

**Fig 3:**
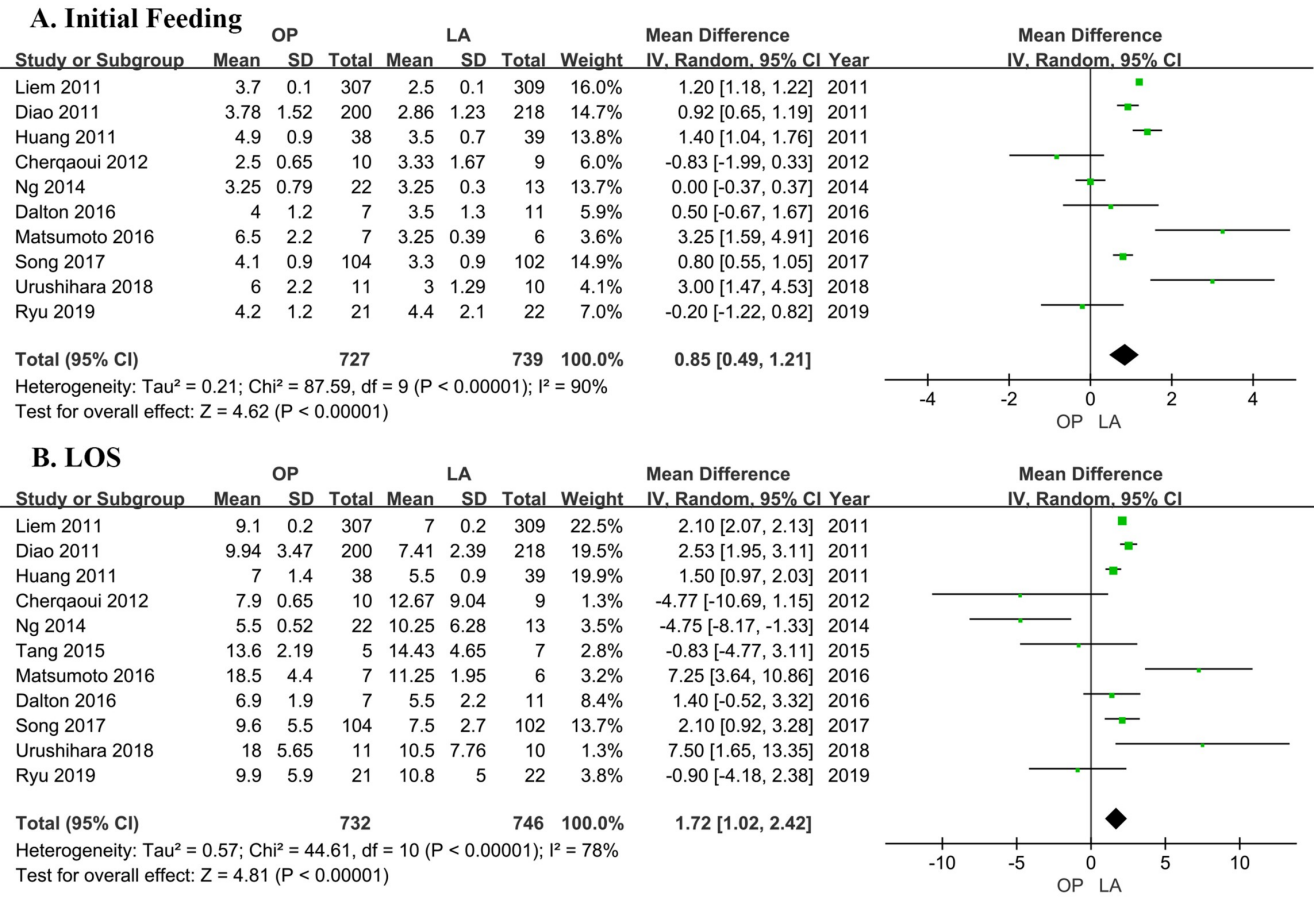
Comparison of the postoperative outcomes of patients who underwent laparoscopic cyst excision and Roux-en-Y hepaticojejunostomy (LA) and open cyst excision and Roux-en-Y hepaticojejunostomy (OP). (A) Initial feeding. (B) Length of hospital stay (LOS).

Ten studies [18–20, 22, 23, 25–29] involved time of initial feeding, including a total of 1466 patients (739 in LA, 727 in OP, Table 2). Seven studies showed the time of initial feeding to be significantly lower in the LA group, whereas 2 showed it to be lower in the OP group. One study reported the time was no significant difference between two groups. Pooled mean difference (MD = 0.85 day; 95% CI = 0.49 to 1.21 days; P<0.00001) indicated statistically shorter time in the LA group. The analysis found statistically significant heterogeneity (I^2^ = 90%), then a random-effect model was adopted (Fig 3A). The results and heterogeneity were not significantly different on sensitivity analysis.

### LOS

Eleven trials [18–23, 25–29] with a total of 1478 patients (746 and 732 who underwent LA and OP, respectively; Table 2) investigated the LOS. Seven studies showed the LOS to be higher in the LA group, whereas 4 showed it to be lower in the OP group. The analysis found statistically significant heterogeneity (I^2^ = 78%), then a random-effect model was adopted. Pooled mean difference (MD = 1.72 days; 95% CI = 1.02 to 2.42 days; P<0.00001) stated statistically shorter time in the LA group (Fig 3B). No differences in the results and no heterogeneity were found on sensitivity analysis.

### Short-term postoperative complications

**Fig 4:**
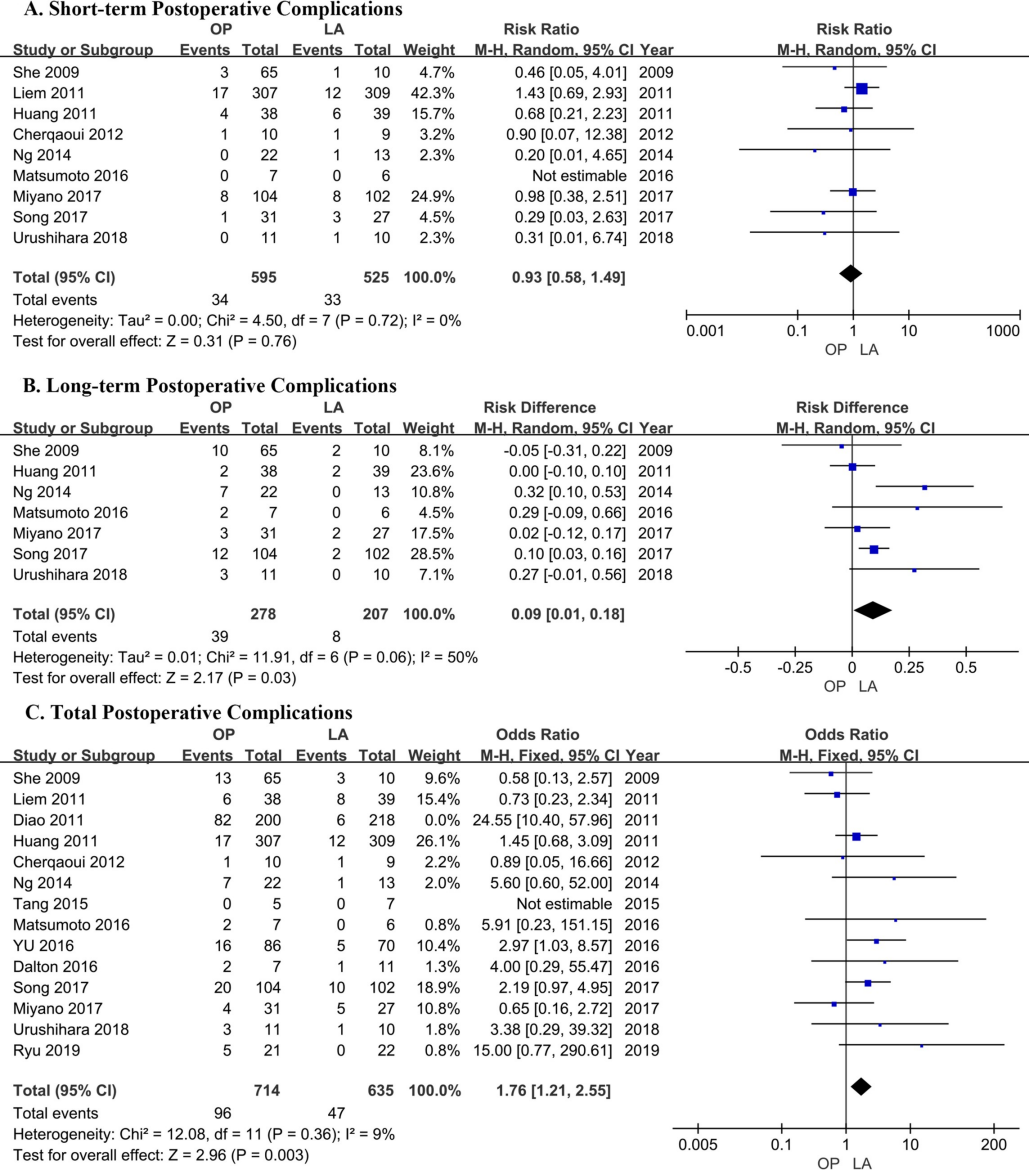
Comparison of the postoperative complications of patients who underwent laparoscopic cyst excision and Roux-en-Y hepaticojejunostomy (LA) and open cyst excision and Roux-en-Y hepaticojejunostomy (OP). (A) Short-term postoperative complications. (B) Long-term postoperative complications. (C) Total postoperative complications.

Nine studies[18–20, 23–26, 29, 30] contributed data, including 1120 patients (525 in the LA group, 595 in the OP group, Table 2). The outcome of meta-analysis (RR = -1.08; 95% CI = -1.72 to -0.67; P = 0.76) stated no statistical difference between the LA and OP groups. Heterogeneity was not significant (P = 0.72, I^2^ = 0%) (Fig 4A).

### Long-term postoperative complications

Seven trails[19, 20, 23, 25, 26, 29, 30] reported long-term postoperative complications, including 485 patients (207 in LA group, 278 in OP group, Table 2). Pooled risk difference (RR = 0.09; 95% CI = 0.01 to 0.18; P = 0.03) stated the morbidity of the long-term postoperative complications was lower in LA group than in OP groups. Heterogeneity was moderate (P = 0.06, I^2^ = 50%), then a random-effect model was adopted (Fig 4B). There were not significantly difference of results and heterogeneity on sensitivity analysis.

### Total postoperative complications

All included studies[17–30] contributed data, including 1767 patients (853 in the LA group, 914 in the OP group; Table 2). Total patient morbidity was 53/853 in the LA group and 178/914 in the OP group. In total postoperative complications, the outcome of meta-analysis (RR = -0.29; 95% CI = -0.40 to -0.21; P<0.00001) showed the total postoperative morbidity was lower in the LA than OP groups. Heterogeneity was high (I^2^ = 74%) (Fig 4C). We conducted a sensitivity analysis by excluding the Diao et al [28]; then the heterogeneity was resolved (P = 0.36, I^2^ = 9%), and the relative risk was also changed (RR = -0.57; 95% CI = -0.83 to -0.39, P = 0.003) (S3 Fig).

### Publication bias

**Fig 5:**
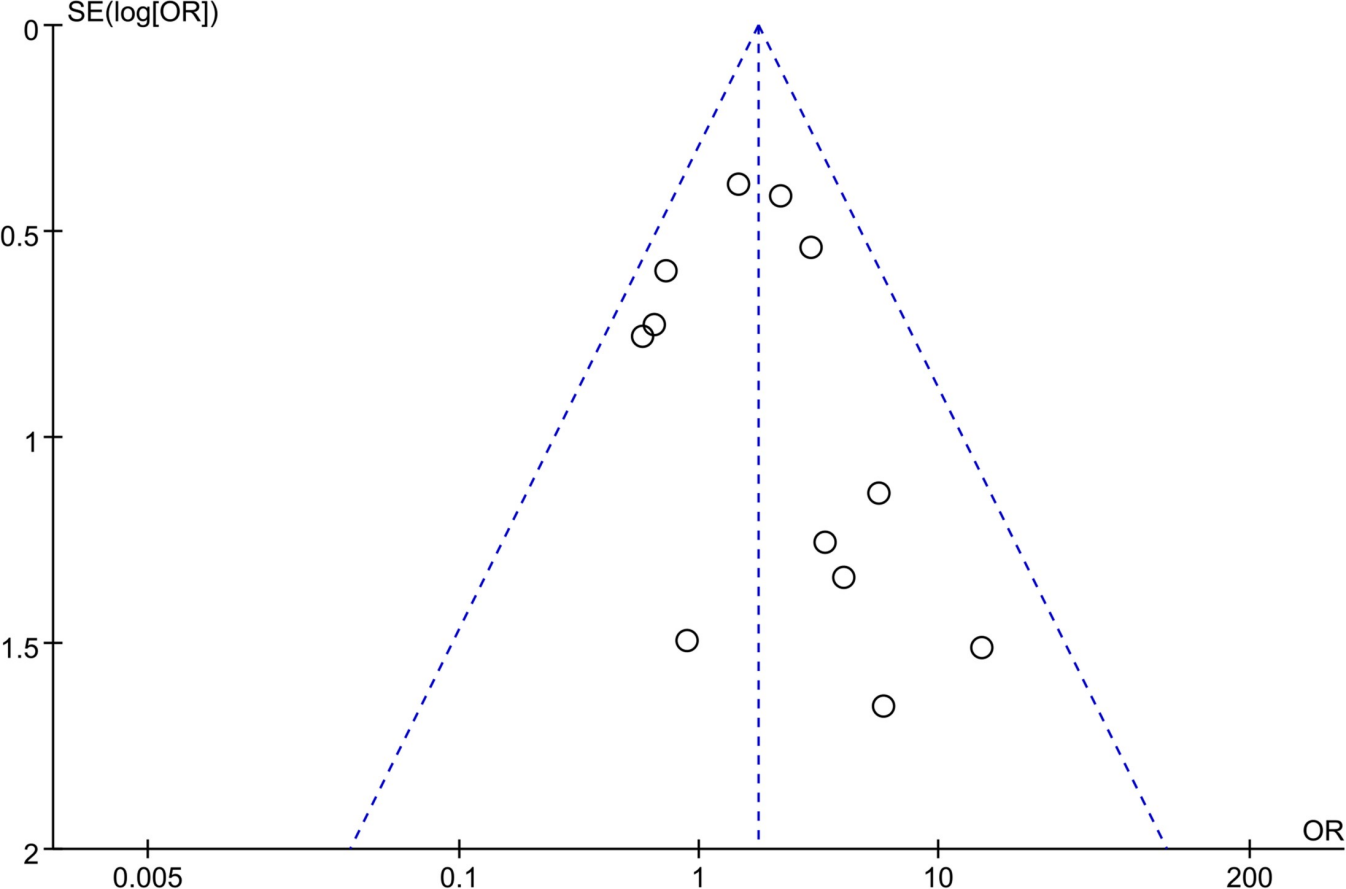
Begg’s funnel plot for assessing publication bias.

Begg’s funnel plot was used to assess any publication bias present in the articles. As shown in the funnel plot of total postoperative complications (Fig 5), no evidence of significant publication bias was found.

## Discussion

The present study was a systematic review and update meta-analysis designed to specifically evaluate the perioperative outcomes of children with CDCs who underwent LA and compare them to those who underwent OP. Overall, the pooled results revealed a significant improvement in long-term and total postoperative complications with LA group, although no significant difference between 2 approach in short-term postoperative complications. Moreover, we found an improvement in LOS with LA, as well as a shorter time of initial feeding, a lower EBIL, and a lower intraoperative blood transfusion volume. However, the operative time was longer in LA group than in OP group. Thus, our findings suggest that the outcomes of LA are at least equivalent to, if no better than, those of OP in children with CDC.

In the present study, operative time was longer in LA group. There is no doubt that the laparoscopic procedure requires specialized curve that may require extensive training in technically challenging and difficult procedures. It was in Wen’s et al. [31] study showed the learning curve of laparoscopic choledochal cyst excision and Roux-en-Y hepaticojejunostomy in children was approximately 37 cases to significantly improve outcomes of operative time, overall postoperative complication rate and the length of hospital stay. Meanwhile, Diao et al. [28] had reported the similar result, in which the number of learning curve was estimate as 35 cases. Liem et al. [18] showed the operating time for LA was comparable to that of OP; meanwhile, they also involved the largest population of patients, which may introduced bias to our pooled results; however, we found no significant change in our results when excluding their study from our analysis. Therefore, we suggest that once the learning curve is achieved, the operating time for laparoscopic procedure might be shortened.

Laparoscopic surgery has the potential to markedly reduce intraoperative blood loss and transfusion, as well as time of initial feeding and LOS in previous studies [10, 11]; these attributes were corroborated in our own study. The heterogeneity of EIBL was high in present study; however, it was decreased by excluding YU et al’s study [17] with the result no change. As YU’s study stated, the mean age of the study was significant older than other study; in addition, the enrolled patients in LA and OP group were symptomatic before surgery. As the previous study [31] showed, with the progress of the disease, the mucosa of the cyst is damaged or even disappeared, the cystic wall become thickened, small vessels develop on the surface of the cyst, and more adhesions develop between the choledochal cyst and surrounding vital structures, such as portal vein and hepatic artery, which may increase the risk and volume of intraoperative bleeding. We assumed that explains the heterogeneity was originated from YU et al’s study. Generally, we use time to commencement of feeds and length of hospitalization as measures of recovery time. Consequently, our findings suggest that the recovery might be faster in LA group compared with OP group. Improvement of intraoperative and postoperative outcomes may be beneficial to the growth of children in physiological.

Although our primary result of this study was that there was no significant difference in short-term postoperative complications between the 2 approaches; however, the other 2 primary results showed a significant improvement in long-term and total postoperative complications among patients with CDC who underwent LA. Short-term complications mean which occur during postoperative hospitalization, including bile leakage, gastrointestinal dysfunction, anastomotic leakage, and wound infection, et al [26]. Meanwhile, long-term complications refer to those happen during the follow-up period, including adhesive ileus, bile duct obstruction, anastomotic stenosis, pancreatitis, and cholangitis, et al. As previous studies showed [8, 32], laparoscopy with its umbilicus-to-hepatic hilum direction of vision provides a better view of the deep anatomic structures, such as hepatic hilum, portal vein, and hepatic arteries. The magnified view from this direction enables meticulous dissection, excision, and ligature; therefore, prevents injuries of the biliary and pancreatic ducts, promotes hemostasis and minimizes blood loss [9], which may reduce complications. This is highly important because most resections are performed on children, which need to be safe and effective. Lower rate of postoperative complications may also be benefit for faster recovery. Moreover, the long-term postoperative complications may have serious impact on growth and development of children. Some sever complications even require surgical interventions [33], which may affect children with more trauma, pain, and scar et al. Nevertheless, in our opinion, one of the key objectives to achieving superior long-term postoperative outcomes with existing treatment modalities is to improve the ability of children to both complete cyst resection and rapidly recover; as such, laparoscopic surgery presents a realistic method to meet this objective.

This meta-analysis has some limitations that should be taken into account when considering the results. Frist, the main limitation of this meta-analysis is the lack of randomized controlled trials. Second, most included studies were conducted in Asian health centers except 2 studies perform in American [22] and German [26], respectively, since CDC is more common in Asian nations. Therefore, there was a risk of selection bias even though such confounders could not be avoided. Third, in some studies, the number of patients was too small, leading to low-power analyses. Lastly, the heterogeneity in some of the results was high. The recommended certainty of some results was low according to the GRADE approach. Thus, some of our results should be interpreted with caution. Overall, additional prospective and multicenter randomized controlled trials with longer follow-up periods are warranted to compare the safety and efficacy of laparoscopic versus open cyst excision and hepaticojejunostomy.

In general, the present study analyzed the perioperative outcomes of LA compared with OP; consequently, it may provide reference basis for surgeon to choose the surgical treatment.

## Conclusion

Laparoscopic open cyst excision and hepaticojejunostomy appears to be effective and safe with intraoperative and postoperative outcomes that are improved to those of open excision in the setting of children with CDCs. With the advantages of less blood loss, smaller trauma, shorter postoperative recovery time, improved cosmetic features, and less incidence of postoperative complications, laparoscopic cyst excision and Roux-en-Y hepaticojejunostomy may become a common procedure for pediatric choledochal cyst in many medical centers.

## Supporting information

S1 Checklist (DOC)

S1 File (PDF)

S2 File (DOCX)

S1 Fig (TIF)

S2 Fig (TIF)

S3 Fig (TIF)
