# Supplementary material for: Comparison of efficacy and safety of laparoscopic excision and open operation in children with choledochal cysts: A systematic review and update meta-analysis
Source: PLoS One. 2020 Sep 28;15(9):e0239857. doi: 10.1371/journal.pone.0239857 (PMC7521726; doi:10.1371/journal.pone.0239857)
Supplement: S1 File — (PDF) [file pone.0239857.s002.pdf]

Systematic review

1. * Review title.

Give the working title of the review, for example the one used for obtaining funding. Ideally the title should state succinctly the interventions or exposures being reviewed and the associated health or social problems. Where appropriate, the title should use the PI(E)COS structure to contain information on the Participants, Intervention (or Exposure) and Comparison groups, the Outcomes to be measured and Study designs to be included.

A systematic review and meta-analysis of laparoscopic versus open excision in children with choledochal cysts

2. Original language title.

For reviews in languages other than English, this field should be used to enter the title in the language of the review. This will be displayed together with the English language title.

3. * Anticipated or actual start date.

Give the date when the systematic review commenced, or is expected to commence.

28/04/2019

4. * Anticipated completion date.

Give the date by which the review is expected to be completed.

31/07/2019

5. * Stage of review at time of this submission.

Indicate the stage of progress of the review by ticking the relevant Started and Completed boxes. Additional information may be added in the free text box provided.
Please note: Reviews that have progressed beyond the point of completing data extraction at the time of initial registration are not eligible for inclusion in PROSPERO. Should evidence of incorrect status and/or completion date being supplied at the time of submission come to light, the content of the PROSPERO record will be removed leaving only the title and named contact details and a statement that inaccuracies in the stage of the review date had been identified.
This field should be updated when any amendments are made to a published record and on completion and publication of the review. If this field was pre-populated from the initial screening questions then you are not able to edit it until the record is published.

The review has not yet started: No

Review stage	Started	Completed
Preliminary searches	Yes	Yes
Piloting of the study selection process	Yes	Yes
Formal screening of search results against eligibility criteria	Yes	No
Data extraction	No	No
Risk of bias (quality) assessment	No	No
Data analysis	No	No

Provide any other relevant information about the stage of the review here (e.g. Funded proposal, protocol not yet finalised).

6. * Named contact.

The named contact acts as the guarantor for the accuracy of the information presented in the register record.

Rui Sun

Email salutation (e.g. "Dr Smith" or "Joanne") for correspondence:

Miss Sun

7. * Named contact email.

Give the electronic mail address of the named contact.

719484238@qq.com

8. Named contact address

Give the full postal address for the named contact.

NO.2, Yabao street, Capital Institute of Pediatrics, Beijing, China

9. Named contact phone number.

Give the telephone number for the named contact, including international dialling code.

008618813019657

10. * Organisational affiliation of the review.

Full title of the organisational affiliations for this review and website address if available. This field may be completed as 'None' if the review is not affiliated to any organisation.

Capital Institute of Pediatrics

Organisation web address:

www.shouer.com.cn

11. * Review team members and their organisational affiliations.

Give the title, first name, last name and the organisational affiliations of each member of the review team. Affiliation refers to groups or organisations to which review team members belong.

Miss Rui Sun. Department of Pediatric Surgery, Capital Institute of Pediatrics

12. * Funding sources/sponsors.

Give details of the individuals, organizations, groups or other legal entities who take responsibility for initiating, managing, sponsoring and/or financing the review. Include any unique identification numbers assigned to the review by the individuals or bodies listed.

None

13. * Conflicts of interest.

List any conditions that could lead to actual or perceived undue influence on judgements concerning the main topic investigated in the review.

None

14. Collaborators.

Give the name and affiliation of any individuals or organisations who are working on the review but who are not listed as review team members.

Professor Mei Diao. Department of Pediatric Surgery, Capital Institute of Pediatrics
Miss Qianqing Li. Department of Pediatric Surgery, Capital Institute of Pediatrics
Professor Long Li. Department of Pediatric Surgery, Capital Institute of Pediatrics
Xiaoliang Liu. Department of Radiation Oncology, Peking Union Medical College Hospital
Na Zhao. Department of Radiology, Fuwai Hospital, State Key Laboratory of Cardiovascular Disease, National Center for Cardiovascular Diseases

15. * Review question.

State the question(s) to be addressed by the review, clearly and precisely. Review questions may be specific or broad. It may be appropriate to break very broad questions down into a series of related more specific questions. Questions may be framed or refined using PI(E)COS where relevant.

What is the efficacy and safety of the laparoscopic surgery for the treatment of choledochal cysts in children compared to open surgery?

16. * Searches.

State the sources that will be searched. Give the search dates, and any restrictions (e.g. language or publication period). Do NOT enter the full search strategy (it may be provided as a link or attachment.)

We will search the following electronic bibliographic databases: PubMed, Embase, The Cochrane Library (Cochrane Database of Systematic Reviews, Cochrane Central Register of Controlled Trials (CENTRAL), Cochrane Methodology Register), ClinicalTrials, and Web of Science (Science and Social Science Citation Index)

The search strategy will include entry words 'Choledochal Cyst' and 'laparoscopic'. The terms will be combined with the Cochrane MEDLINE filter for controlled trials of interventions. The search strategy for PubMed is available in the published protocol. The search terms will be adapted for use with other bibliographic databases in combination with database-specific filters for controlled trials, where these are available.

There will be English reports only. There will be no published time restrictions.

17. URL to search strategy.

Give a link to a published pdf/word document detailing either the search strategy or an example of a search strategy for a specific database if available (including the keywords that will be used in the search strategies), or upload your search strategy.Do NOT provide links to your search results.

https://www.crd.york.ac.uk/PROSPEROFILES/137474_STRATEGY_20190531.pdf

Alternatively, upload your search strategy to CRD in pdf format. Please note that by doing so you are consenting to the file being made publicly accessible.

Do not make this file publicly available until the review is complete

18. * Condition or domain being studied.

Give a short description of the disease, condition or healthcare domain being studied. This could include health and wellbeing outcomes.

Choledochal cyst (CDC) is a rare congenital cystic dilation of the biliary tract. It accounts for approximately 1 % of all benign biliary diseases. CDC requires surgical intervention in order to avoid complications such as cholangitis, perforation, liver failure and malignancy.

19. * Participants/population.

Give summary criteria for the participants or populations being studied by the review. The preferred format includes details of both inclusion and exclusion criteria.

Inclusion: Children with Choledochal cysts.

Exclusion: Adults (over 18 years of age)

20. * Intervention(s), exposure(s).

Give full and clear descriptions or definitions of the nature of the interventions or the exposures to be reviewed.

The intervention is the laparoscopic surgery for the treatment of choledochal cysts in children

21. * Comparator(s)/control.

Where relevant, give details of the alternatives against which the main subject/topic of the review will be compared (e.g. another intervention or a non-exposed control group). The preferred format includes details of both inclusion and exclusion criteria.

The comparator is the open surgery for the treatment of choledochal cysts in children

22. * Types of study to be included.

Give details of the types of study (study designs) eligible for inclusion in the review. If there are no restrictions on the types of study design eligible for inclusion, or certain study types are excluded, this should be stated. The preferred format includes details of both inclusion and exclusion criteria.

We will include the observational studies (including cohort and case–control studies) to assess the efficacy and safety of the laparoscopic treatment.

23. Context.

Give summary details of the setting and other relevant characteristics which help define the inclusion or exclusion criteria.

Studies report the 2 surgical techniques for the treatment of choledochal cyst. Studies compare the 2 surgical techniques. Studies include at least one of the main outcomes, used for data analysis.

24. * Main outcome(s).

Give the pre-specified main (most important) outcomes of the review, including details of how the outcome is defined and measured and when these measurement are made, if these are part of the review inclusion criteria.

Those reponsible for the included studies will be asked to supply the following outcomes of 2 surgery group: operative time; hospital stay; postoperative complications.

Timing and effect measures

Perioperative period

25. * Additional outcome(s).

List the pre-specified additional outcomes of the review, with a similar level of detail to that required for main outcomes. Where there are no additional outcomes please state 'None' or 'Not applicable' as appropriate to the review

Intraoperative blood transfusion; Diameter of cyst

Timing and effect measures

Perioperative period

26. * Data extraction (selection and coding).

Describe how studies will be selected for inclusion. State what data will be extracted or obtained. State how this will be done and recorded.

Two investigators (Rui Sun and Na Zhao) independently assessed selected studies according to NOS and extracted the following information: first author, year of publication, study type, mean age, number of population, and main outcomes of interest (operative time, hospital stay, intraoperative blood loss, recovery of bowel function, intraoperative blood transfution, and complications, etc.). We use Excel to record the results. We use the Review manager 5.3 to conduct the study.

27. * Risk of bias (quality) assessment.

Describe the method of assessing risk of bias or quality assessment. State which characteristics of the studies will be assessed and any formal risk of bias tools that will be used.

Two review authors will independently assess the quality and the risk of bias according to the NOS standard. Disagreements between the review authors over the risk of bias in particular studies will be resolved by discussion, with involvement of a third review author where necessary.

28. * Strategy for data synthesis.

Provide details of the planned synthesis including a rationale for the methods selected. This **must not be generic text** but should be **specific to your review** and describe how the proposed analysis will be applied to your data.

We will provide a narrative synthesis of the findings from the included studies, structured around the type of intervention, target population characteristics, type of outcome and intervention content. Statistical analysis of dichotomous variables was performed using the odds ratio (OR) as the summary statistic, while continuous variables were analyzed using the weighted mean difference (MD). For both variables, 95 % confidence intervals (CI) were reported. Heterogeneity was assessed using the I^2 statistic.

29. * Analysis of subgroups or subsets.

State any planned investigation of ‘subgroups’. Be clear and specific about which type of study or participant will be included in each group or covariate investigated. State the planned analytic approach.

This is a qualitative synthesis and while subgroup analyses may be undertaken it is not possible to specify the groups in advance

30. * Type and method of review.

Select the type of review and the review method from the lists below. Select the health area(s) of interest for your review.

Type of review

Cost effectiveness
No

Diagnostic
No

Epidemiologic
No

Individual patient data (IPD) meta-analysis
No

Intervention
No

Meta-analysis
Yes

Methodology
No

Narrative synthesis
Yes

Network meta-analysis
No

Pre-clinical
No

Prevention
No

Prognostic
No

Prospective meta-analysis (PMA)
No

Review of reviews
No

Service delivery
No

Synthesis of qualitative studies

No

Systematic review
Yes

Other
No

Health area of the review

Alcohol/substance misuse/abuse
No

Blood and immune system
No

Cancer
No

Cardiovascular
No

Care of the elderly
No

Child health
No

Complementary therapies
No

Crime and justice
No

Dental
No

Digestive system
No

Ear, nose and throat
No

Education
No

Endocrine and metabolic disorders
No

Eye disorders
No

General interest
No

Genetics
No

Health inequalities/health equity
No

Infections and infestations
No

International development
No

Mental health and behavioural conditions
No

Musculoskeletal
No

Neurological

No

Nursing
No

Obstetrics and gynaecology
No

Oral health
No

Palliative care
No

Perioperative care
No

Physiotherapy
No

Pregnancy and childbirth
No

Public health (including social determinants of health)
No

Rehabilitation
No

Respiratory disorders
No

Service delivery
No

Skin disorders
No

Social care
No

Surgery
No

Tropical Medicine
No

Urological
No

Wounds, injuries and accidents
No

Violence and abuse
No

31. Language.

Select each language individually to add it to the list below, use the bin icon to remove any added in error.
English

There is an English language summary.

32. Country.

Select the country in which the review is being carried out from the drop down list. For multi-national collaborations select all the countries involved.

China

33. Other registration details.

Give the name of any organisation where the systematic review title or protocol is registered (such as with The Campbell Collaboration, or The Joanna Briggs Institute) together with any unique identification number assigned. (N.B. Registration details for Cochrane protocols will be automatically entered). If extracted data will be stored and made available through a repository such as the Systematic Review Data Repository (SRDR), details and a link should be included here. If none, leave blank.

34. Reference and/or URL for published protocol.

Give the citation and link for the published protocol, if there is one

Give the link to the published protocol.

Alternatively, upload your published protocol to CRD in pdf format. Please note that by doing so you are consenting to the file being made publicly accessible.

No I do not make this file publicly available until the review is complete

Please note that the information required in the PROSPERO registration form must be completed in full even if access to a protocol is given.

35. Dissemination plans.

Give brief details of plans for communicating essential messages from the review to the appropriate audiences.

Do you intend to publish the review on completion?

Yes

36. Keywords.

Give words or phrases that best describe the review. Separate keywords with a semicolon or new line. Keywords will help users find the review in the Register (the words do not appear in the public record but are included in searches). Be as specific and precise as possible. Avoid acronyms and abbreviations unless these are in wide use.

Systematic review; meta-analysis; Laparoscopic; Open; Choledochal cyst; children.

37. Details of any existing review of the same topic by the same authors.

Give details of earlier versions of the systematic review if an update of an existing review is being registered, including full bibliographic reference if possible.

38. * Current review status.

Review status should be updated when the review is completed and when it is published. For newregistrations the review must be Ongoing.
Please provide anticipated publication date

Review_Ongoing

39. Any additional information.

Provide any other information the review team feel is relevant to the registration of the review.

40. Details of final report/publication(s).

This field should be left empty until details of the completed review are available.

NHS
National Institute for
Health Research

Give the link to the published review.
