# Supplementary material for: Comparison of efficacy and safety of laparoscopic excision and open operation in children with choledochal cysts: A systematic review and update meta-analysis
Source: PLoS One. 2020 Sep 28;15(9):e0239857. doi: 10.1371/journal.pone.0239857 (PMC7521726; doi:10.1371/journal.pone.0239857)

Choledochal Cyst 主题词

自由词

Choledochal Cysts

Cyst, Choledochal

Cysts, Choledochal

Bile Duct Cysts

Bile Duct Cyst

Cyst, Bile Duct

Cysts, Bile Duct

Duct Cyst, Bile

Duct Cysts, Bile

Choledochocele

Choledochoceles

Congenital Choledochal Cyst

Choledochal Cyst, Congenital

Choledochal Cysts, Congenital

Congenital Choledochal Cysts

Cyst, Congenital Choledochal

Cysts, Congenital Choledochal

Choledochal Cyst, Type V

Intrahepatic Choledochal Cyst

Choledochal Cyst, Intrahepatic

Choledochal Cysts, Intrahepatic

Cyst, Intrahepatic Choledochal

Cysts, Intrahepatic Choledochal

Intrahepatic Choledochal Cysts

Choledochal Cyst, Type III

Choledochal Cyst, Type IV

Multiple Choledochal Cysts

Choledochal Cyst, Multiple

Choledochal Cysts, Multiple

Cyst, Multiple Choledochal

Cysts, Multiple Choledochal

Multiple Choledochal Cyst

Choledochal Cyst, Type I

Cyst, Common Bile Duct

Cysts, Common Bile Duct

Common Bile Duct Cyst

Choledochal Cyst, Type II

Choledochal Diverticulum

Choledochal Diverticulums

Diverticulum, Choledochal

Diverticulums, Choledochal

Choledochal Cyst, Diverticulum

Choledochal Cysts, Diverticulum

Diverticulum Choledochal Cyst

Diverticulum Choledochal Cysts

Laparoscopy 主题词

自由词

Laparoscopies

Celioscopy

Celioscopies

Peritoneoscopy

Peritoneoscopies

Surgical Procedures, Laparoscopic

Laparoscopic Surgical Procedure

Procedure, Laparoscopic Surgical

Procedures, Laparoscopic Surgical

Surgery, Laparoscopic

Laparoscopic Surgical Procedures

Laparoscopic Surgery

Laparoscopic Surgeries

Surgeries, Laparoscopic

Laparoscopic Assisted Surgery

Laparoscopic Assisted Surgeries

Surgeries, Laparoscopic Assisted

Surgery, Laparoscopic Assisted

Surgical Procedure, Laparoscopic

General Surgery 主题词

Surgery, General

Surgery

Pubmed 检索结果：249篇

检索式：

((("Laparoscopy"[Mesh]) OR (((((((((((((((((((Surgical Procedure, Laparoscopic[Title/Abstract]) OR Laparoscopies[Title/Abstract]) OR Celioscopy[Title/Abstract]) OR Celioscopies[Title/Abstract]) OR Peritoneoscopy[Title/Abstract]) OR Peritoneoscopies[Title/Abstract]) OR Surgical Procedures, Laparoscopic[Title/Abstract]) OR Laparoscopic Surgical Procedure[Title/Abstract]) OR Procedure, Laparoscopic Surgical[Title/Abstract]) OR Procedures, Laparoscopic Surgical[Title/Abstract]) OR Surgery, Laparoscopic[Title/Abstract]) OR Laparoscopic Surgical Procedures[Title/Abstract]) OR Laparoscopic Surgery[Title/Abstract]) OR Laparoscopic Surgeries[Title/Abstract]) OR Surgeries, Laparoscopic[Title/Abstract]) OR Laparoscopic Assisted Surgery[Title/Abstract]) OR Laparoscopic Assisted Surgeries[Title/Abstract]) OR Surgeries, Laparoscopic Assisted[Title/Abstract]) OR Surgery, Laparoscopic Assisted[Title/Abstract]))) AND (("Choledochal Cyst"[Mesh]) OR (((((((((((((((((((((((((((((((((((((((((((((Diverticulum Choledochal Cysts[Title/Abstract]) OR Choledochal Cysts[Title/Abstract]) OR Cyst, Choledochal[Title/Abstract]) OR Cysts, Choledochal[Title/Abstract]) OR Bile Duct Cysts[Title/Abstract]) OR Bile Duct Cyst[Title/Abstract]) OR Cyst, Bile Duct[Title/Abstract]) OR Cysts, Bile Duct[Title/Abstract]) OR Duct Cyst, Bile[Title/Abstract]) OR Duct Cysts, Bile[Title/Abstract]) OR Choledochocele[Title/Abstract]) OR Choledochoceles[Title/Abstract]) OR Congenital Choledochal Cyst[Title/Abstract]) OR Choledochal Cyst, Congenital[Title/Abstract]) OR Choledochal Cysts, Congenital[Title/Abstract]) OR Congenital Choledochal Cysts[Title/Abstract]) OR Cyst, Congenital Choledochal[Title/Abstract]) OR Cysts, Congenital Choledochal[Title/Abstract]) OR Choledochal Cyst, Type V[Title/Abstract]) OR Intrahepatic Choledochal Cyst[Title/Abstract]) OR Choledochal Cyst, Intrahepatic[Title/Abstract]) OR Choledochal Cysts, Intrahepatic[Title/Abstract]) OR Cyst, Intrahepatic Choledochal[Title/Abstract]) OR Cysts, Intrahepatic Choledochal[Title/Abstract]) OR Intrahepatic Choledochal Cysts[Title/Abstract]) OR Choledochal Cyst, Type III[Title/Abstract]) OR Choledochal Cyst, Type IV[Title/Abstract]) OR Multiple Choledochal Cysts[Title/Abstract]) OR Choledochal Cyst, Multiple[Title/Abstract]) OR Choledochal Cysts, Multiple[Title/Abstract]) OR Cyst, Multiple Choledochal[Title/Abstract]) OR Cysts, Multiple Choledochal[Title/Abstract]) OR Multiple Choledochal Cyst[Title/Abstract]) OR Choledochal Cyst, Type I[Title/Abstract]) OR Cyst, Common Bile Duct[Title/Abstract]) OR Cysts, Common Bile Duct[Title/Abstract]) OR Common Bile Duct Cyst[Title/Abstract]) OR Choledochal Cyst, Type II[Title/Abstract]) OR Choledochal Diverticulum[Title/Abstract]) OR Choledochal Diverticulums[Title/Abstract]) OR Diverticulum, Choledochal[Title/Abstract]) OR Diverticulums, Choledochal[Title/Abstract]) OR Choledochal Cyst, Diverticulum[Title/Abstract]) OR Choledochal Cysts, Diverticulum[Title/Abstract]) OR Diverticulum Choledochal Cyst[Title/Abstract]))

Embase检索结果：275篇

Cochrane 检索结果：10篇

ClinicalTrails 检索结果：0篇

Web of scince 检索结果：220篇

检索式：TS=(Choledochal Cyst* OR Cyst*, Choledochal OR Bile Duct Cyst* OR Cyst*, Bile Duct OR Duct Cyst*, Bile OR Choledochocele* OR Congenital Choledochal Cyst* OR Choledochal Cyst*, Congenital OR Cyst*, Congenital Choledochal OR Choledochal Cyst, Type V OR Intrahepatic Choledochal Cyst* OR Choledochal Cyst*, Intrahepatic OR Cyst*, Intrahepatic Choledochal OR Choledochal Cyst, Type III OR Choledochal Cyst, Type IV OR Multiple Choledochal Cyst* OR Choledochal Cyst*, Multiple OR Cyst*, Multiple Choledochal OR Choledochal Cyst, Type I OR Cyst*, Common Bile Duct OR Common Bile Duct Cyst* OR Choledochal Cyst, Type II OR Choledochal Diverticulum* OR Diverticulum*, Choledochal OR Choledochal Cyst*, Diverticulum OR Diverticulum Choledochal Cyst*)

TS=(Laparoscopy OR Laparoscopies OR Celioscopy OR Celioscopies OR Peritoneoscopy OR Peritoneoscopies OR Surgical Procedures, Laparoscopic OR Laparoscopic Surgical Procedure OR Procedure*, Laparoscopic Surgical OR Surgery, Laparoscopic OR Laparoscopic Surgical Procedures OR Laparoscopic Surgery OR Laparoscopic Surgeries OR Surgeries, Laparoscopic OR Laparoscopic Assisted Surgery OR Laparoscopic Assisted Surgeries OR Surgeries, Laparoscopic Assisted OR Surgery, Laparoscopic Assisted OR Surgical Procedure, Laparoscopic)

文献总数：755

其中：重复文献：201（删除）注：有500篇是错误文献

Records identified through datebase searching (n=754)

Pubmed: 249

Embase: 275

Cochrane library: 10

Web of Science: 220

ClinicalTrials.gov: 0

Records after duplicates removed (n=553)

Records after reading titles and abstracts

(n=28)

Full texts excluded(n= 12),

with reasons

-not RCT or CCT

-not knee joint

Records excluded (n=526)

Letter: 3

Case report: 72

Not releated study: 320

Meeting abstract: 7

Study of Adults: 22

Not comparative study: 58

Review and meta analysis: 33

No Abstract: 11

Full texts assesed for eligibility

(n=19)

Studies included in quantitative synthesis

(n=7)

主题词：Robotics

Remote Operations (Robotics)

Operation, Remote (Robotics)

Operations, Remote (Robotics)

Remote Operation (Robotics)

Telerobotics

Soft Robotics

Robotic, Soft

Robotics, Soft

Soft Robotic

检索出只有12篇，暂时不可行

Fig.1A Operative time
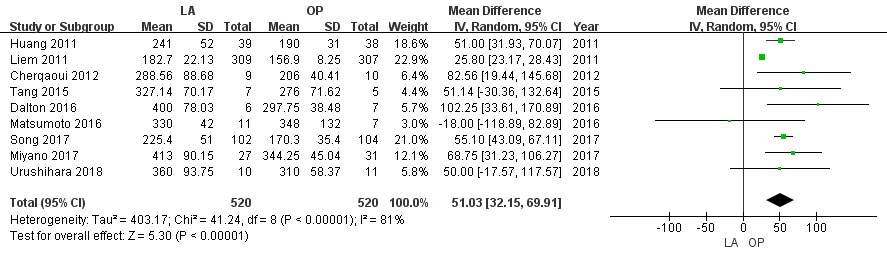


Fig.1B Intraoperative bleeding


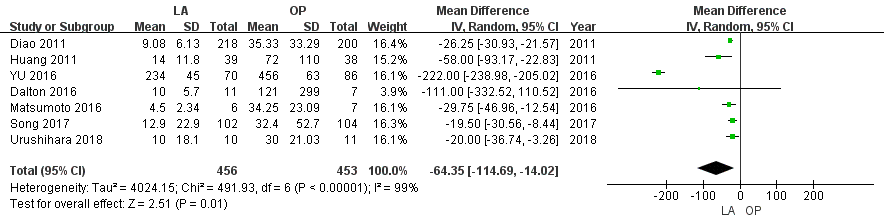


Fig.1C Intraoperative blood transfusion


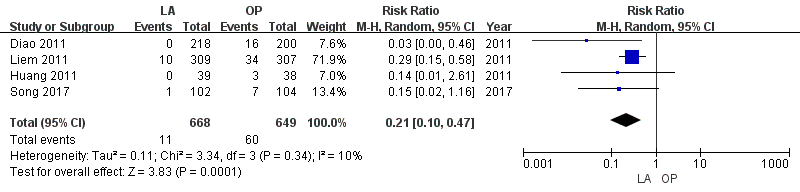


Fig.1D Recovery of bowel function


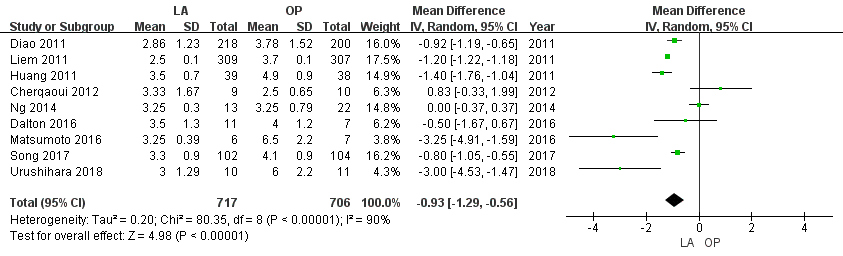


Fig.1E Hospital stay


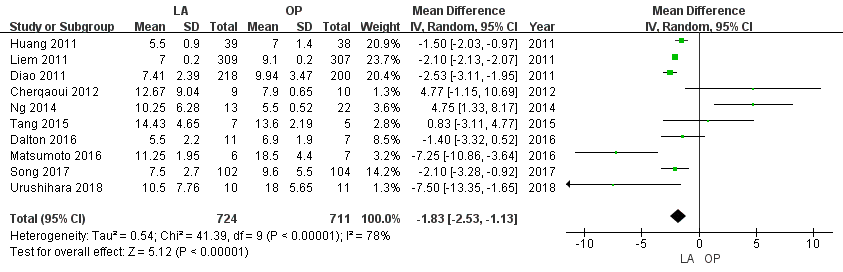


Fig.2B Early postoperative complications


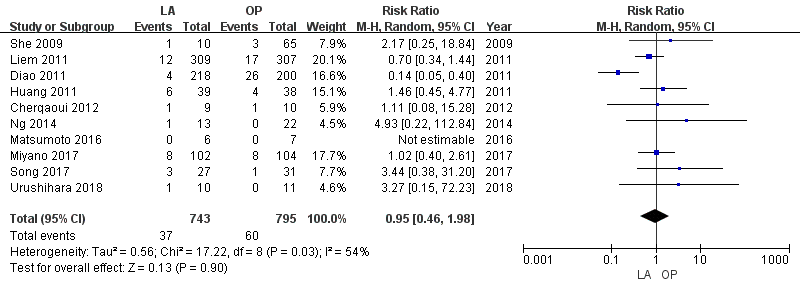


Fig.2C Long-term postoperative complications


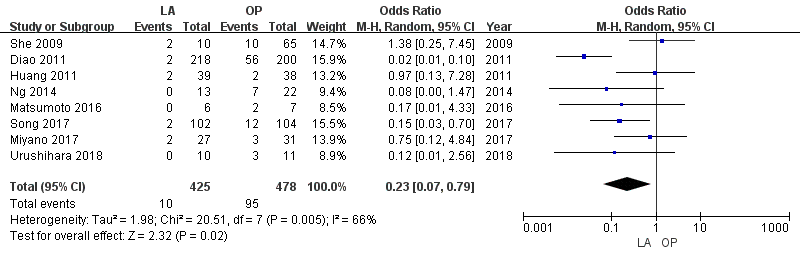


Fig.2D Total postoperative complications


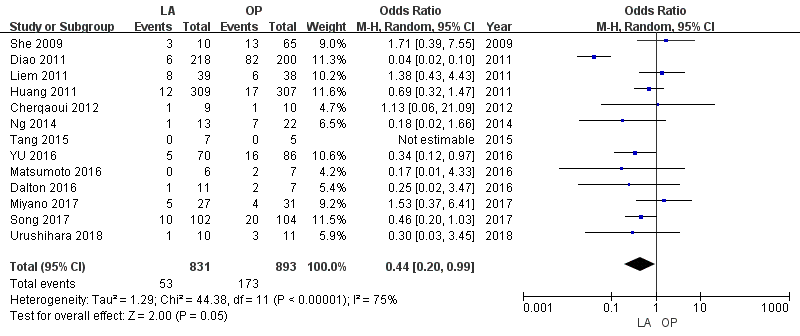


Fig.3A Operative time


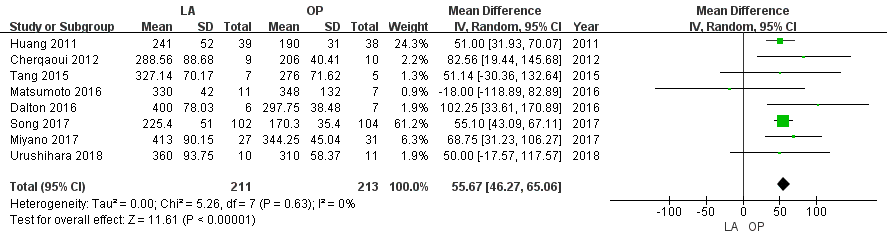


Fig.3B Intraoperative bleeding


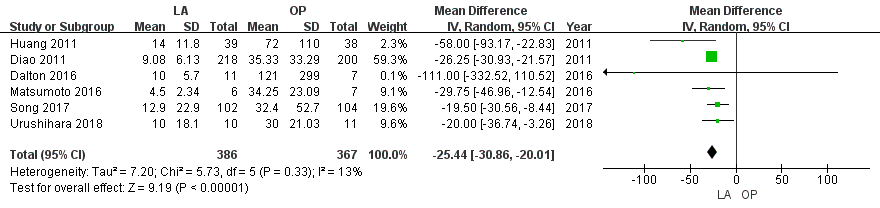


Fig.3C Total postoperative complications


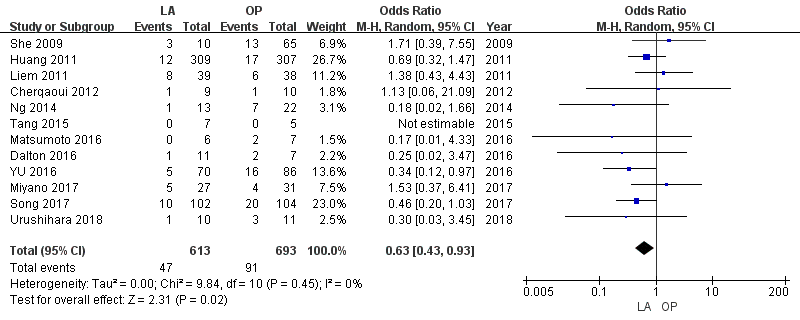

Supplement: S2 File — (DOCX) [file pone.0239857.s003.docx]
