# Supplementary figures and images for: Comparison of efficacy and safety of laparoscopic excision and open operation in children with choledochal cysts: A systematic review and update meta-analysis
Source: PLoS One. 2020 Sep 28;15(9):e0239857. doi: 10.1371/journal.pone.0239857 (PMC7521726; doi:10.1371/journal.pone.0239857)

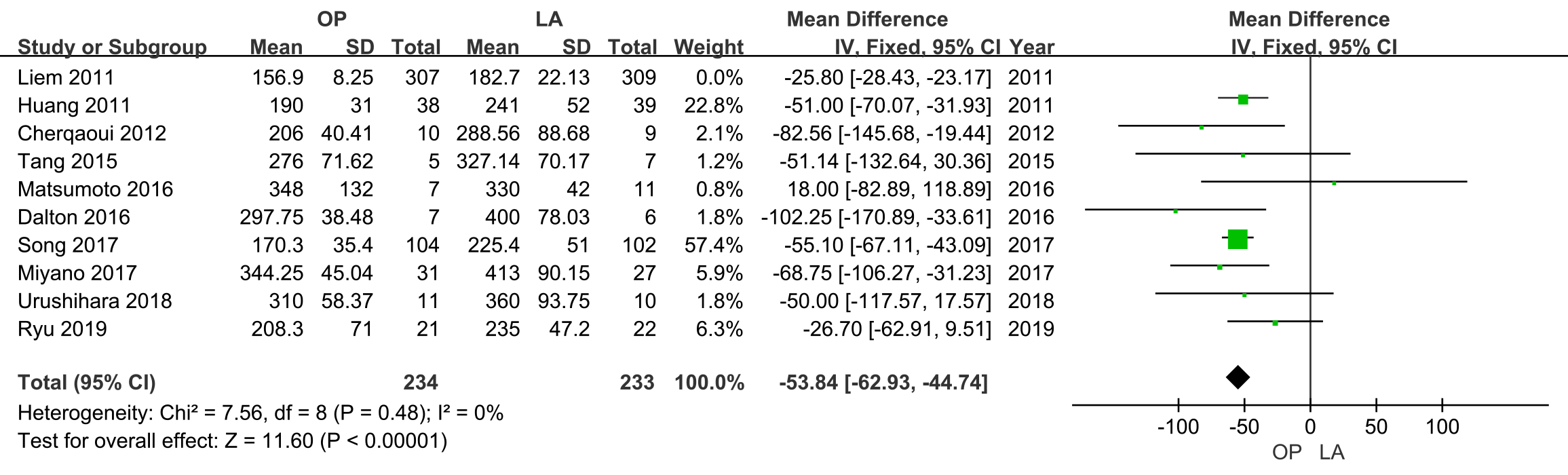

Supplement: S1 Fig — (TIF) [file pone.0239857.s004.tif]

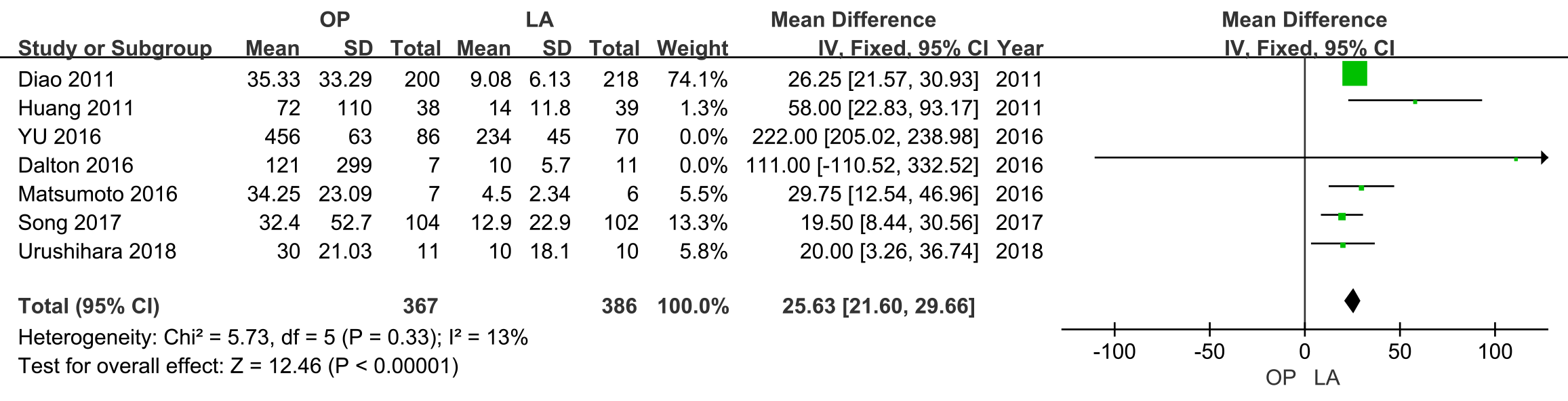

Supplement: S2 Fig — (TIF) [file pone.0239857.s005.tif]

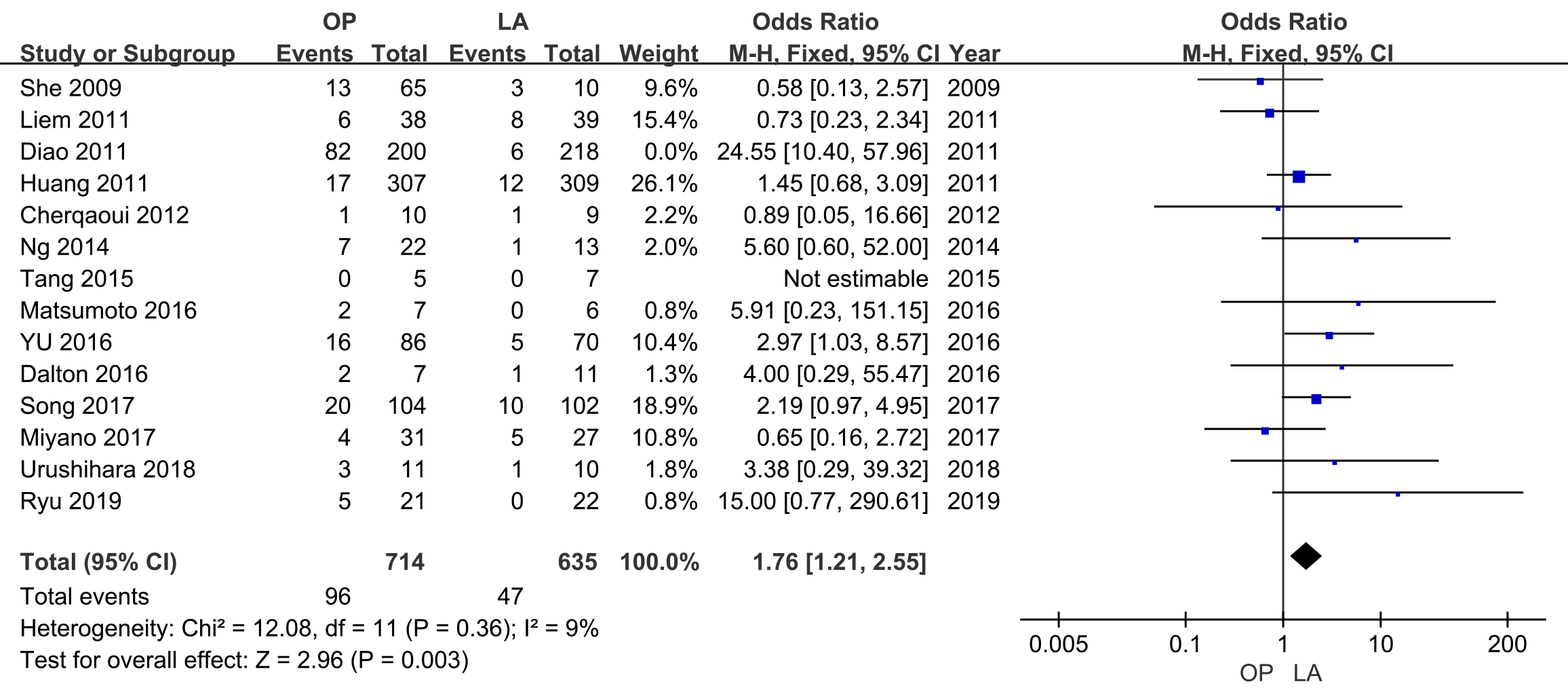

Supplement: S3 Fig — (TIF) [file pone.0239857.s006.tif]
